# Evolution of the *CBL* and *CIPK* gene families in *Medicago*: genome-wide characterization, pervasive duplication, and expression pattern under salt and drought stress

**DOI:** 10.1186/s12870-022-03884-3

**Published:** 2022-11-03

**Authors:** Xiao-Xia Zhang, Xiao-Long Ren, Xiao-Tong Qi, Zhi-Min Yang, Xiao-Lei Feng, Tian Zhang, Hui-Jie Wang, Peng Liang, Qi-Ying Jiang, Wen-Jun Yang, Yuan Fu, Min Chen, Zhi-Xi Fu, Bo Xu

**Affiliations:** 1 State Key Laboratory of Systematic and Evolutionary Botany, Institute of Botany, Chinese Academy of Sciences, Beijing, 100093 China; 2 University of Chinese Academy of Sciences, Beijing, 100049 China; 3 Zhangjiakou Academy of Agricultural Sciences, Zhangjiakou, 075000 China; 4 Institute of Botany, Jiangsu Province and Chinese Academy of Sciences, Nanjing, 210014 China; 5 College of Life Sciences, Sichuan Normal University, Chengdu, 610101 China

**Keywords:** Calcineurin B-like protein (CBL), CBL-interacting protein kinase (CIPK), Gene family evolution, Duplicate genes, Stress response, *Medicago*

## Abstract

**Background:**

Calcineurin B-like proteins (CBLs) are ubiquitous Ca^2+^ sensors that mediate plant responses to various stress and developmental processes by interacting with CBL-interacting protein kinases (CIPKs). CBLs and CIPKs play essential roles in acclimatization of crop plants. However, evolution of these two gene families in the genus *Medicago* is poorly understood.

**Results:**

A total of 68 *CBL* and 135 *CIPK* genes have been identified in five genomes from *Medicago*. Among these genomes, the gene number of *CBL*s and *CIPK*s shows no significant difference at the haploid genome level. Phylogenetic and comprehensive characteristic analyses reveal that *CBL*s and *CIPK*s are classified into four clades respectively, which is validated by distribution of conserved motifs. The synteny analysis indicates that the whole genome duplication events (WGDs) have contributed to the expansion of both families. Expression analysis demonstrates that two *MsCBL*s and three *MsCIPK*s are specifically expressed in roots, mature leaves, developing flowers and nitrogen fixing nodules of *Medicago sativa* spp. *sativa*, the widely grown tetraploid species. In particular, the expression of these five genes was highly up-regulated in roots when exposed to salt and drought stress, indicating crucial roles in stress responses.

**Conclusions:**

Our study leads to a comprehensive understanding of evolution of *CBL* and *CIPK* gene families in *Medicago*, but also provides a rich resource to further address the functions of CBL-CIPK complexes in cultivated species and their closely related wild relatives.

**Supplementary Information:**

The online version contains supplementary material available at 10.1186/s12870-022-03884-3.

## Background

The calcium ion (Ca^2+^) is acknowledged as a ubiquitous second messenger, which is perceived by sensor proteins mediating the signal transduction pathways. In plants, calcineurin B-like (CBL) proteins are one of the major Ca^2+^ sensors, which are recognized and bound specifically by CBL-interacting protein kinases (CIPKs), forming the CBL-CIPK complex to decode and transduce Ca^2+^ signals [1, 2]. CBL-CIPK signaling pathways play critical roles in perception and response to unfavourable environmental stimuli, such as salt and drought [3, 4]. In addition, the CBL-CIPK complex is also involved in plant growth and development, including seedling growth, flower development, pollen germination and pollen tube growth, and root development [1–6]. Throughtout plant life cyle, CBLs and CIPKs perform key function to maintain the balance between plant optimal growth and yield production under stress conditions [3, 7]. Therefore, it is important to understand the functions of CBLs and CIPKs for genetic improvement of crop plants with enhanced fitness and production.

CBL proteins harbor four conserved α-helix-loop-α-helix structures, designated as EF-hand motifs, which are responsible for Ca^2+^ binding [8–10]. CBL proteins are divided into three types based on the amino acid sequences occurred at the N-terminus. These sequences guide CBL proteins to distinct subcellular compartments, thus further determining their function. Type I CBL proteins are detected at the plasma membrane with a dual lipid modification motif (MGCXXS/T) [8, 11]. Type II CBL proteins are characterized by a conserved sequence (MSQCXDGXKHXCXSXXXCF), which is tonoplast targeting sequence (TTS) [11, 12]. Type III CBL proteins contain a transmembrane helical region (TM helix), and their localizations can be at tonoplast or plasma membrane [8, 11]. Additionally, a highly conserved PFPF/FPSF motif at the C-terminus of CBL proteins possesses a serine residue that is responsible for the phosphorylation of CBL proteins by CIPKs [13, 14].

CIPK proteins bind to CBLs through their NAF/FISL motif in the regulatory domain at C-terminus [15, 16]. This motif is highlighted with 6 conserved amino acid residues, including Asn (N), Ala (A), Phe (F), Ile (I), Ser (S) and Leu (L), which are essential for the interaction of CIPK proteins with CBLs [11, 15, 16]. Next to NAF/FISL motif, a protein phosphatase interaction motif (PPI) is commonly found, but it is not well conserved among CIPK proteins [17, 18]. It has been suggested that PPI mediates the interaction of CIPKs with abscisic acid insensitive (ABI) or protein phosphatase 2C (PP2C) proteins [7]. In addition, CIPK proteins also are characterized by a kinase/catalytic domain at the N-terminus [19]. This domain contains a phosphate-binding site that accounts for the CIPK phosphotransferase activity. In general, the kinase/catalytic domain remains blocked by the regulatory domain of CIPK proteins, and interaction with CBLs is required for its activation [17]. Therefore, CIPK proteins are recruited by CBLs in a calcium-dependent manner [20, 21].

As an important forage crop, *M. sativa*, is widely cultivated in the world. Cultivated *M. sativa* supplies as a major source of nitrogen for livestock animals, thus, is referred to as the queen of forages. However, the *M. sativa* forage is unable to supply the dramatic increase in demand for livestock production in current society. Exploitation of genetic variations underlying agronomic traits in wild relatives closed to the cultivated *Medicago* is a key to speed up *M. sativa* breeding. The culitivted *M. sativa* is a complex, including diploids (2n = 2 ×  = 16, e.g. *M. sativa* ssp. *caerulea*) [22] and tetraploids (2n = 4 ×  = 32, *M. sativa* spp. *sativa*) [23]. Wild species closely related to *M. sativa* are found in the genus of *Medicago*, such as *M. truncatula* (a legume model plant) [24], *M. ruthenica* [25, 26], and *M. polymorpha* [27]. As with *M. sativa* spp. *caerulea*, these three species are diploid plants [28]. Ploidy level variations represent the abundance of genetic diversity in *Medicago*, providing a extensive genetic pool for *Medicago* species to withstand environmental changes as well as for *M. sativa* breeding [28]*.* Recently, rapid increase of genome and transcriptome data from species in the genus *Medicago* facilitates researches to address the fundamental mechanisms, which in return will accelerate *M. sativa* breeding with molecular approaches [29, 30].

Identification of significant genetic variations underlying the key agronomical traits provides potential applications to develop *M. sativa* cultivars with enhanced resistance to drought, salt and other stress [3, 31], which can adapt to various environments and grow in a wide range. Since the first discovery of CBL and CIPK proteins as critical components in salt overly sensitive pathway (SOS) mediating salt stress response in *Arabidopsis thaliana* [32], these two families have been reported in many plant species by genome-wide studies, including 10 *CBL*s and 26 *CIPK*s from *A. thaliana*, 7 *CBL*s and 20 *CIPK*s from *Triticum aestivum*, and 9 *CBL*s and 35 *CIPK*s in *Oryza sativa* [10, 33, 34]. CBL-CIPK signaling pathways are essential for plants to survive in the rapid changes of external environments [7, 35]. The CBL-CIPK complexes serve as a key regulatory hub in transduction of Ca^2+^ signals to control the uptake of essential nutrient ions, such as K^+^, H^+^, Mg^2+^, Na^+^, Fe^2+^, NO_3_^−^, NH_4_^+^, as well as to maintain the ion homeostasis under stress conditions by directly regulating the activities of ion channels and transporters at plasma membrane and tonoplast [36, 37]. In *A. thaliana*, AtCBL4/SOS3, AtCBL10, AtCIPK21, and AtCIPK24/SOS2 function under high-salt condition. AtCBL1, AtCBL9 and AtCIPK23 are reported to respond to drought stress [3, 38–45]. In rice, many *CBL*s and *CIPK*s are induced by drought treatment, and overexpression of *OsCIPK3*, *OsCIPK12* or *OSCIPK15* promotes drought and salt tolerance respectively [34, 46]. The maize plants with loss function of *ZmCBL4*, *ZmCBL8* or *ZmCBL24* are sensitive when subjected to salt treatment [47]. In apple (*Malus domestica*), overexpression of *MdCIPK22* enhances drought tolerance by promoting the sugar accumulation in vacuoles [48]. As stated, *CBL* and *CIPK* family genes are promising candidates to be utilized for *M. sativa* breeding and improvement. Recent study identified *CBL* genes and *CIPK*s in *M. truncatula* and *M. sativa* [49], however, it is still poorly understood how these two gene families have evolved in the genus *Medicago* and its close relatives.

In the current study, we identified CBL and CIPK proteins from 5 *Medicago* species/subspecies and their close relatives, and gene structure and motif conservation were extensively examined. To better understand the evolutionary histories of CBL and CIPK families in the genus *Medicago*, phylogenetic relationship, gene duplication, and synteny were comprehensively investigated. In addition, expression of *CBL* and *CIPK* genes in response to salt and drought stress was analyzed in *M. sativa* spp. *sativa*. Our study sheds in-depth insights into evolution of CBL and CIPK families in the genus *Medicago*, and provide a rich genetic resource that could be further investigated and utilized to promote the resistance to salt and drought stress in *M. sativa* and its close relatives.

## Results

### Genome-wide identification of *CBL* and *CIPK* genes in *Medicago*

**Fig. 1:**
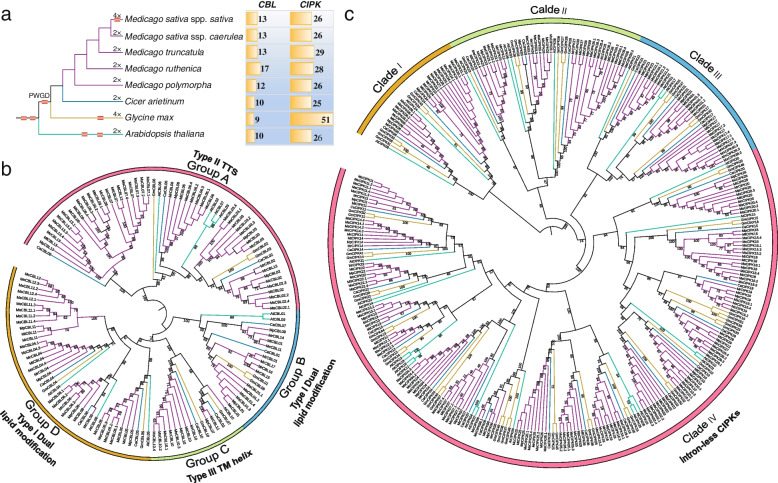
Phylogenetic relationships of the *CBL* and *CIPK* genes. **a** Numbers of gene members from *CBL* and *CIPK* families in studied genomes. Red box on branch of species tree represents the whole genome duplication event. PWGD: the ancestral Papilionoideae whole-genome duplication event. The gene number was calculated at the haploid genome level (one set of chromosomes) of each species. 2 × : diploid, 4 × : tetraploid. **b** The phylogeny of *CBL* gene family. **c** The phylogeny of *CIPK* family. Branches in different colors indicate different taxa groups

**Fig. 2:**
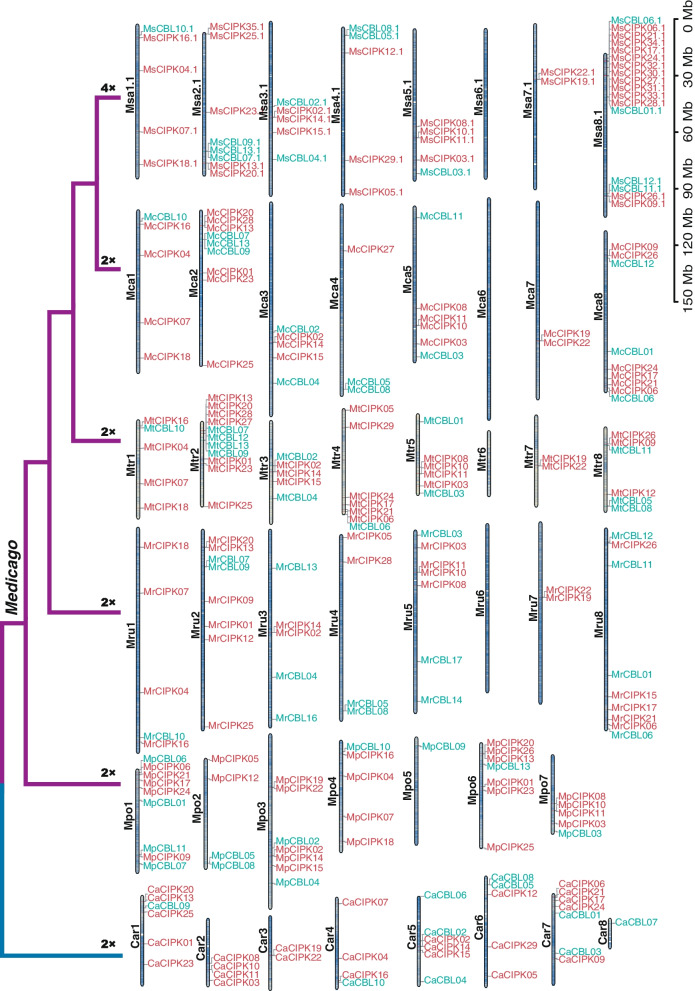
The chromosomal localization of the *CBL* and *CIPK* genes in *Medicago* and *C. arietinum*. The ribbons represent chromosomes from each genome. Note that several genes are not anchored to chromosomes due to the quality of genome assembly. *C. arietinum* is selected as outgroup. The chromosome number is denoted on the left of each chromosome. All chromosomes are demonstrated at the same scale in megabase

*CBL* and *CIPK* genes were reported in *M. sativa* and *M. truncatula* [49], however, due to requirement of consistent standard for identification of *CBL*s and *CIPK*s from selected species in our study, we re-identified them in these two species. In total, at haploid genome level (*n* = 1 ×  = 8), the model legume plant *M. truncatula* contains 13 *CBL*s and 29 *CIPK*s, 13 *CBL*s and 26 *CIPK*s were obtained from *M. sativa* ssp. *caerulea,* 17 *CBL*s and 28 *CIPK*s were detected in *M. ruthenica*, 12 *CBL*s and 26 *CIPK*s were identified from *M. polymorpha*, and 10 *CBL*s and 25 *CIPK*s were retrieved from *Cicer arietinum* (Fig. 1a). As expected, the cultivated autotetraploid *M. sativa* ssp. *sativa* (2n = 4 ×  = 32) possesses more gene members than diploid species in *Medicago* genus, and a total of 49 *CBL*s (Additional file 1: Table S1) and 107 *CIPK*s (Additional file 2: Table S2) were identified. However, at the haploid genome level in tetraploid *M. sativa* ssp. *sativa*, 13 *CBL*s and 26 *CIPK*s were obtained, with the same gene numbers in its diploid progenitor *M. sativa* ssp. *caerulea*, which is consistent with previous report [49]. Moreover, similar numbers of *CBL*s or *CIPK*s were observed in five *Medicago* species/subspecies at the haploid genome level. Collectively, we identified 68 *CBL*s and 135 *CIPK*s in five genomes from the genus *Medicago* at the level of haploid genome (Fig. 1a). All these genes were designated according to their homologs in *A. thaliana*, and the chromosomal locations and phylogenetic relationships were also taken into consideration (Figs. 1b, c and 2; Additional file 1: Table S1; Additional file 2: Table S2; Additional file 3: Fig. S1).

The physicochemical properties of CBLs and CIPKs identified in this study were examined, including number of amino acids, protein size, isoelectric point (pI), and molecula weight (MW). The length of CBL protein sequences ranges from 143 to 421 amino acids (AAs) with the 229 AAs as an average (Additional file 1: Table S1). The pI varies from 4.46 to 6.35, and their MW falls within the range of 16.54 kDa to 46.59 kDa (Additional file 1: Table S1). The CIPK proteins are 132–518 AAs in length with the 435 AAs as an average, and their pI ranges from 4.84 to 9.32, and the MW of CIPKs ranges from 15.13 kDa to 58.03 kDa (Additional file 2: Table S2). The variations in length, pI, and MW suggest that the function of CBLs and CIPKs are highly diversified, and may be involved in various processes associated with growth, development, and stress responses.

The identified genes are physically anchored on the chromosomes in five genomes. Overall, *CBL* and *CIPK* genes are unevenly distributed on chromosomes from each *Medicago* genome (Fig. 2). For instance, Msa08.1 contains the most number of *CBL*s and *CIPK*s in *M. sativa* spp. *sativa*, whereas no *CBL* or *CIPK* gene is found on Msa06.1 (Fig. 2). Similar patterns are also exhibited in diploid genomes, including *M. sativa* ssp. *caerulea, M. truncatula*, and *M. ruthenica*. It is noted that most *CBL* and *CIPK* genes preferentially sit in the chromosomal regions with high gene density.

### Phylogenetic relationships of *CBL* and *CIPK* genes in *Medicago*

A dataset with 133 CBL protein sequences was collected to generate the phylogenetic tree, including 114 CBL proteins from *Medicago* and *C. arietinum*, and 19 CBL proteins previously reported in *A. thaliana* and *Glycine max* (Additional file 4: Table S3) [50]*.* The CBL proteins are assigned to four groups (Group A, B, C and D) as specified by their membrane targeting motifs (Fig. 1b). Based on the phylogeny of CBL family with strong support, it was found that the Group A is comprised of all Type II CBL proteins sharing the TTS motifs (Additional file 4: Fig. S1). The members from Group B are Type I CBL proteins harboring a dual lipid modification motif, including AtCBL4, AtCBL8 and AtCBL5, which are localized at the plasma membrane (Additional file 5: Fig. S2). Similarly, proteins in the Group D are also belonged to the Type I CBL, such as AtCBL1 and AtCBL9 (Additional file 5: Fig. S2). The Type III CBLs are characterized by a single transmembrane helix at the N-terminus, forming the well-supported Group C (Additional file 5: Fig. S2).

All 318 CIPK protein sequences were collected to perform phylogenetic analysis (Additional file 6: Table S4), and they are classified into four clades with high bootstrap values (Fig. 1c, Clade I, II, III and IV). The *CIPK*s from Clade I, II and III are intron-rich (Fig. 1c), among which, the Clade I is the most basal group including proteins homologous to AtCIPK24/SOS2, such as McCIPK08 and MrCIPK24. In addition, the Clade IV is well supported as a monophyletic branch comprising the intron-less *CIPK* genes in *Medicago* (Fig. 1c).

### Gene structure and motif conservation of *CBL*s and *CIPK*s

**Fig. 3:**
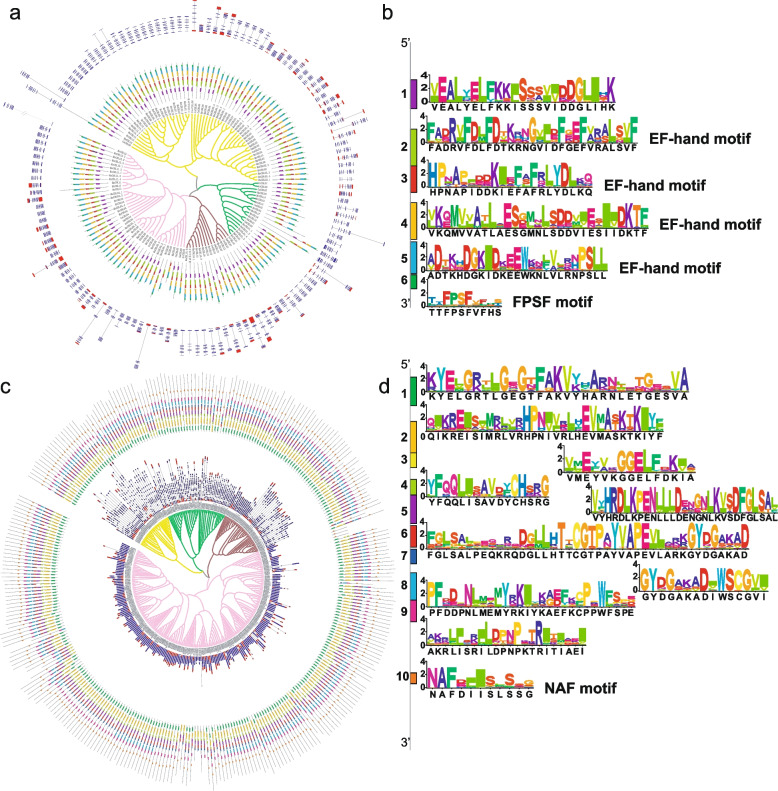
The relatively conserved gene structures and motifs in *CBL* and *CIPK* gene families. **a** The gene structures and motifs of *CBL* genes. The circle outside shows gene structures, and the one inside exhibits 6 motifs characterized. The branches in four different colors indicate four groups of *CBL* genes respectively.** b** Seq logos of conserved motifs for CBL proteins. **c** The gene structures and motifs of *CIPK* genes. The circle outside shows 10 motifs identified, and the one inside represents gene structures. The branches in four different colors indicate four clades of *CIPK* genes respectively. **d** Seq logos of conserved motifs for CIPK proteins

Gene structure and motif conservation provide key information to understand the gene family evolution and functional diversification among its members. All *CBL* genes identified in *Medicago* possess a number of introns varying from 7 to 10 (Fig. 3a). Six conserved motifs were characterized by MEME-motif scanning program in CBL proteins (Fig. 3a, b). The sequence logos were displayed and referred to four EF-hand motifs and one FPSF motif respectively (Fig. 3b). The subcellular localization motifs were further divided into three types (Additional file 5: Fig. S2), which is in line with the evolutionary relationship of *CBL* genes (Fig. 1b).

The intron numbers of *CIPK* genes lays in the range of 0 to 14. Based on this observation, *CIPK* genes are divided into two groups, intron-rich group and intron-less group, respectively (Fig. 3c). The intron-rich *CIPK* genes all belong to the Clade I, II and III, whereas all intron-less *CIPK* genes are exclusively found in the Clade IV (Fig. 1c). There are ten motifs identified in CIPK proteins. Notably, the NAF motif at the C-terminus is highly conserved in all CIPKs, which can be considered as the feature motif for CIPK family (Fig. 3c, d). Taken together, these results show that the gene structure and motif of CBLs and CIPKs are well conserved in *Medicago*, indicating their evolutionary relationships and molecular functions.

### Duplication, synteny, and expansion pattern of *CBL* and *CIPK* genes

**Fig. 4:**
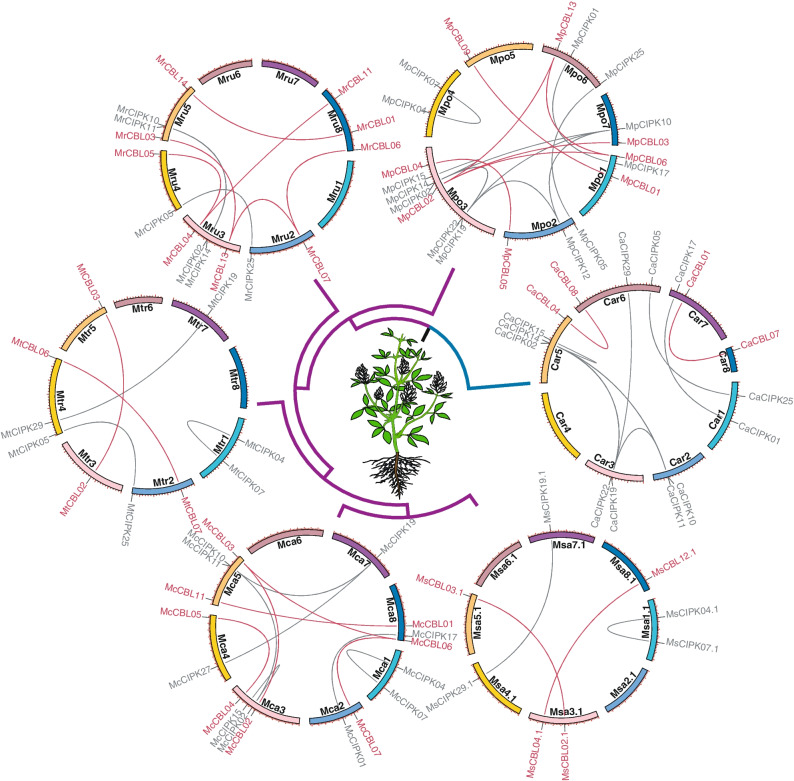
The intraspecies syntenic relationship of *CBL* and *CIPK* genes in *Medicago* and *C. arietinum*. The duplicate gene pairs retained after PWGD event are revealed. *C. arietinum* is taken as outgroup. The lines in red and gray represent the syntenic relationship of *CBL* and *CIPK* gene pairs, respectively

To investigate how these two families expanded in *Medicago*, duplication pattern of *CBL*s and *CIPK*s was examined. Based on the synteny within each species and synonymous substitution (*Ks*) value of duplicate gene pairs, it was found that 23 pairs of *CBL*s and 28 pairs of *CIPK*s from five *Medicago* genomes and *C. arietinum* have been retained since the shared WGD event by all Papilionoideae species (x = 8) (Fig. 1a and Additional file 7: Table S5), which is known as PWGD event [51]. The detailed syntenic relationships of intra-species were further investigated (Fig. 4). After the PWGD, 2 pairs of *CBL*s and 2 pairs of *CIPK*s in *M. sativa* ssp. *sativa*, 5 pairs of *CBL*s and 5 pairs of *CIPK*s in *M. sativa* ssp. *caerulea*, 2 pairs of *CBL*s and 3 pairs of *CIPK*s in *M. truncatula,* 6 pairs of *CBL*s and 2 pairs of *CIPK*s in *M. ruthenica,* 6 pairs of *CBL*s and 9 pairs of *CIPK*s in *M. polymorpha*, and 2 pairs of *CBL*s and 7 pairs of *CIPK*s in *C. arietinum* have been preserved (Fig. 4)*.*

**Fig. 5:**
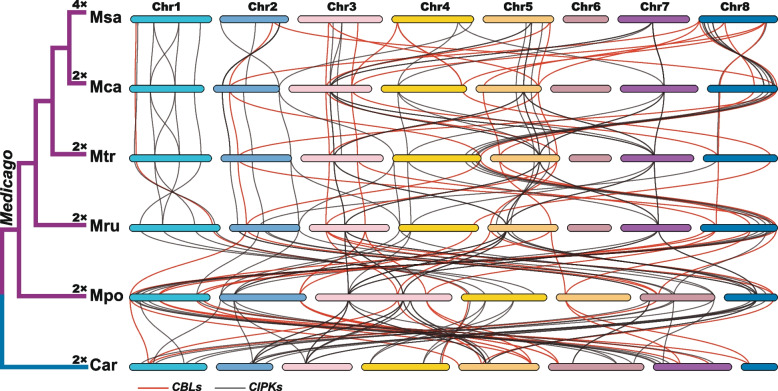
The interspecies syntenic relationship of *CBL* and *CIPK* genes in *Medicago* and *C. arietinum*. The collinearity of *CBL* and *CIPK* orthologous genes are indicated using red and gray lines

To better understand the evolutionary relationships of both gene families, the inter-species synteny was analyzed among the six genomes, including five *Medicago* species/subspecies plus *C. arietinum*. Consequently, syntenic gene pairs were extensively present among these six genomes, such as 20 *CBL* and 36 *CIPK* syntenic gene pairs between *M. sativa* ssp. *sativa* and *M. sativa* ssp. *caerulea*, 15 *CBL* and 39 *CIPK* syntenic gene pairs between *M. sativa* ssp. *caerulea* and *M. truncatula*, 14 *CBL* and 35 *CIPK* syntenic gene pairs between *M. truncatula* and *M. ruthenica*, 21 *CBL* and 42 *CIPK* syntenic gene pairs between *M. ruthenica* and *M. polymorpha*, and 19 *CBL* and 48 *CIPK* syntenic gene pairs between *M. polymorpha* and *C. arietinum* (Fig. 5)*.* The wide presence of syntenic connections among these six genomes from Papilionoideae indicates that the PWGD has contributed significantly to the expansion of both gene families.

The cultivated *M. sativa* spp. *sativa* is an autotetraploid species displaying tetrasomic inheritance (2n = 4 ×  = 32). Following the PWGD, its diploid ancestor underwent an independent lineage-specific autotetraploidy event, referred to Msa-WGD event. To visualize the expansion patterns of *CBL* and *CIPK* genes in *M. sativa* spp. *sativa*, a total of 49 *CBL*s (Additional file 1: Table S1) and 107 *CIPK*s (Additional file 2: Table S2) were identified (Additional file 3: Fig. S1). It was found that 25 pairs of *CBL*s and 54 pairs of *CIPK*s survived after two-round WGDs in the tetraploid *M. sativa* spp. *sativa* (Additional file 8: Fig. S3). We further checked the synonymous substitution (*Ks*) values for all syntenic duplicate gene pairs (Additional file 7: Table S5), and found that there are 19 pairs of *CBL*s and 39 pairs of *CIPK*s preserved after Msa-WGD (Additional file 8: Fig. S3).

In addition to syntenic duplicates, 3 pairs of *CBL*s and 5 pairs of *CIPK*s were found survived after a number of independent recent tandem duplication events in *M. sativa* spp. *sativa* (Additional file 3: Fig. S1). Among them, the tandem duplicate gene *MsCBL09*/*MsCBL13*/*MsCBL07* have been retained on each chromosome set of tetrasomic inheritance genome, indicating that the tandem duplication occurred before Msa-WGD (Additional file 3: Fig. S1). The tandem duplications of *MsCBL11.1*/*MsCBL12.1*, *MsCBL05.1*/*MsCBL08.1*, and *MsCIPK26*/*MsCIPK09* show the similar expansion pattern to that of *MsCBL09.1*/*MsCBL13.1*/*MsCBL07.1* (Additional file 3: Fig. S1). By contrast, the tandem duplications of *MsCIPK35.1*/*MsCIPK25.1*, *MsCIPK37.3*/*MsCIPK40.3*/*MsCIPK38.3*, *MsCIPK36.2*/*MsCIPK05.2*, and *MsCIPK32.1*/*MsCIPK30.1*/*MsCIPK27.1*/*MsCIPK31.1*/*MsCIPK33.1* are only present on one chromosome, but their paralogs are not detected on the other three homologous chromosomes (Additional file 3: Fig. S1). These results indicate that the tandem duplications took place on individual chromosomes independently, which is later than Msa-WGD.

### Expression profile of *CBL* and *CIPK* genes in *M. sativa* spp. *sativa*

The expression of *CBL* and *CIPK* genes was examined using public RNA-seq data of six tissues from *M. sativa* spp. *sativa*, including roots, mature leaves, developing flowers, elongating stem internodes, post-elongating stem internodes, and nitrogen fixing nodules [29]. Most of *CBL*s and *CIPK*s are expressed with distinct abundance in tested tissues, except for 3 *CBL*s (*MsCBL07/09/13*) and 1 *CIKP* (*MsCIPK18*), whose expressions are too low to be detected. There are 9 *CBL*s (*MsCBL03.1/01.1/02.1/11.1/12.1/06.1/10.1/08.1/04.1*) (Additional file 9: Fig. S4) and 10 *CIPK*s (*MsCIPK06.1/16.1/19.1/25.1/35.1/23.1/04.1/03.1/11.1/12.1*) (Additional file 10: Fig. S5) are relatively highly expressed. Tissue-specific expression was also observed. For instance, the transcripts of *MsCBL04* and *MsCBL08* are preferentially enriched in nitrogen fixing nodules and roots respectively (Additional file 9: Fig. S4), and *MsCIPK12/21/34* (Additional file 10: Fig. S5) are exclusively expressed in flowers. These results suggest that *CBL* and *CIPK* genes are widely associated with *M. sativa* spp. *sativa* development, and may function in a tissue-specific manner.

To better understand their roles in stress responses, we further investigated the expression of *CBL* and *CIPK* genes in roots from *M. sativa* spp. *sativa* when exposed to the salt and drought treatment, by taking advantage of the public RNA-seq data [30]. When treated by 250 mM NaCl (salt) or 400 mM mannitol (drought), 8 *CBL*s (*MsCBL12.1/04.1/03.1/01.1/08.1/11.1/02.1/06.1*) (Additional file 11: Fig. S6) and 12 *CIPK*s (*MsCIPK06.1/25.1/35.1/23.1/16.1/03.1/11.1/12.1/04.1/07.1/08.1/19.1*) (Additional file 12: Fig. S7) are highly up-regulated. Particularly, the transcripts of *MsCBL04.1*, *MsCBL012.1*, *MsCIPK06.1*, *MsCIPK25.1*, and *MsCIPK35.1* are dramatically accumulated along the treatments (Additional files 9, 10, 11 and 12: Fig. S4-S7), which were further confirmed by quantitative RT-PCR analysis (Additional file 13: Fig. S8). These genes were remarkably upregulated when exposed to the salt and drought after 12 h (Additional file 13: Fig. S8). Our observations suggested crucial roles of these genes in mediating responses to salt and drought stress. In summary, these results provide a pool of candidate genes to elucidate the functions of *CBL* and *CIPK* genes, which could be potentially adopted to improve cultivated *Medicago* plants with enhanced salt or drought tolerance.

## Discussion

*Medicago* species have been widely cultivated as forage for livestock in the world. With the increasing genome sequencing data from *Medicago* species and their close relatives, it provides a great opportunity to investigate the fundamental questions in *Medicago* biology, which in return will aid the cultivated *Medicago* breeding and improvement [22, 23, 25–27, 52, 53]. As a versatile intracellular messenger, roles of Ca^2+^ in salt and drought stress responses in plants have been extensively studied in last two decades [1, 54]. The CBL-CIPK complex functions as a key regulatory hub to balance the growth and stress responses in environmental adaptations by plants [36]. Thus, the signal-specific responses of CBLs and CIPKs are of great interest to develop resilient and nutrient-efficient crops. Although *CBL* and *CIPK* genes had been identified in *M. sativa* and *M. truncatula* [49], it is still unclear how these two gene families have evolved in *Medicago*. Here, we conducted a genome-wide systemic analysis of *CBL* and *CIPK* gene families in *Medicago* and its relatives, including evolutionary relationships, chromosomal localizations, gene structures, motifs, and duplication patterns, which can contribute to the further studies of CBLs and CIPKs in cultivated *Medicago* species.

### Pervasive duplication events contributed to the expansion of *CBL* and *CIPK* families

Both *CBL* and *CIPK* gene families have expanded independently in multiple land plant lineages [11, 50]. WGD is able to generate massive duplicated genes instantly, leading to expansion of gene families [50, 55, 56]. Our results support that the PWGD is one of the most important events driving the expansion of *CBL* and *CIPK* gene families in *Medicago* and its relative, *C. arietinum* (Fig. 4). Notably, we also found that the numbers of *CBL* and *CIPK* genes show no significant variation among the studied genomes from five *Medicago* species/subspecies, *C. arietinum*, and *A. thaliana* at haploid level (*n* = 1 ×) (Fig. 1a). Despite a couple of rounds of WGDs occurred during evolution of these species [51, 52, 57], the numbers of *CBL* and *CIPK* genes stay relatively constantly in the tested diploid species. This pattern could be explained by the gene balance hypothesis (GBH) [58], in which copy numbers of genes encoding proteins for assembly of macromolecular complex are under selection to follow the stoichiometric dosage for their proper function [50, 59, 60]. CBL proteins interact with CIPKs to form the CBL-CIPK complexes in a dosage-balance-sensitive manner (Additional file 14: Figure S9), and the copy numbers of these genes are constrained more strictly than those of dosage-insensitive genes [50, 61, 62]. Furthermore, the GBH could also account for the observation that the retained duplicates of *CBL* and *CIPK* genes are barely derived from the tandem duplication event in *M. sativa* spp. *sativa* (Additional file 3: Figure S1; Additional file 8: Figure S3). Tandem duplication takes place by chance, however, the increased gene copies lead to disruption of their natural balance in the formation of CBL-CIPK complex [57, 58, 62]. As a result, the extra copies of duplicated genes would be prone to be eliminated during the process of subsequent evolution [57, 58, 62].

The number of gene family members in autotetraploid can be considered approximately as the sum of numbers from four sets of monoploid genome. For instance, in the autotetraploid *M. sativa* ssp. *sativa*, the number of *CBL*s to *CIPK*s is 49:107 (2n = 4 ×  = 32), roughly a four-fold increase when compared with that in monoploid, which is 13:26 (*n* = 1 ×  = 8) (Fig. 1a; Additional file 1: Table S1; Additional file 2: Table S2). By contrast, the allotetraploids exhibit a non-additive pattern. In the allotetraploid *G. max*, the member of *CBL*s to *CIPK*s is 9:51 (*n* = 2 ×  = 20) (Fig. 1a). These results indicate the presence of different mechanisms underlying the retention/loss of duplicated genes between autotetraploids and allotetraploids, and that the duplicated genes tend to be retained in autotetraploids rather than in allotetraploids [50, 58–62].

PWGD, Msa-WGD, and small-scale tandem duplication have played core roles in the expansion of *CBL* and *CIPK* gene families in *Medicago*, among which the WGDs are major contributors. The stoichiometric dosage balance underlying proper assembly of obligate CBL-CIPK complex is an important evolutionary trajectory [50, 57]. Autotetraploids are much more plastic to cope with the multiple gene copies than diploids and allotetraploids, probably by success in fixation of additive superior genes or alleles. Thus, our study provides an important theoretical guidance for the autopolyploid breeding in crops.

### *CBL*s and *CIPK*s are relatively conserved in *Medicago*

In addition to reveal the evolution of gene family, construction of phylogenetic relationship has been widely employed to predict gene functions as well as to identify key features of proteins. Phylogenetic analysis shows that both *CBL*s and *CIPK*s are grouped into four clades, and the gene structure and motif are relatively conserved in *Medicago* (Fig. 3a, c), which is consistent with our previous research in other land plant species [50]. The variation in physicochemical properties suggests that the functions of CBLs and CIPKs are highly diversified, and may be involved in various biological processes in tissue differentiations and stress responses. The motif analysis shows that the vast majority of CBLs contain a well conserved FPSF motif, whilst all CIPKs are highly conserved in NAF motif at the C-terminal. The NAF and FPSP motifs are features for CIPKs and CBLs respectively, probably due to that CIKPs require the NAF motif to specifically bind to CBLs with FPSF motif, which is essential for proper interaction to form the CBL-CIPK complex [7, 63].

Chromosomal localization analysis reveals that the Chromosome 6 harbors no *CBL* or *CIPK* genes, and the Chromosome 7 possesses 2 *CIPK*s in tested *Medicago* species/subspecies, except *M. polymorpha* (Fig. 2). Uneven distribution of gene family members on chromosomes is a universal phenomenon that is closely associated with the evolution and genetic variation of plant species. For instance, there are more numbers of *CBL* and *CIPK* genes on the chromosome Mpo3 in *M. polymorpha* than those on the corresponding chromosomes (Mru3, Mtr3, Mca3 and Msa3.1) in other *Medicago* species/subspecies tested in this study (Fig. 2), which may be caused by the species-specific chromosomal fusion events [27]. In *M. polymorpha*, Mpo3 is a fused chromosome that is equivalent to the Chromosome 3 and 7 in *M. ruthenica, M. truncatula*, and *M. sativa* [27]. Moreover, extensive interspecies synteny between 5 *Medicago* species/subspecies and *C. arietinum* was revealed (Fig. 5), suggesting that the orthologous genes of *CBL*s and *CIPK*s are conserved respectively, despite the occurrence of recombination and presence of structural variations on their chromosomes during evolution of these species [64].

### Roles of CBLs and CIPKs in development and abiotic stress tolerance

**Fig. 6:**
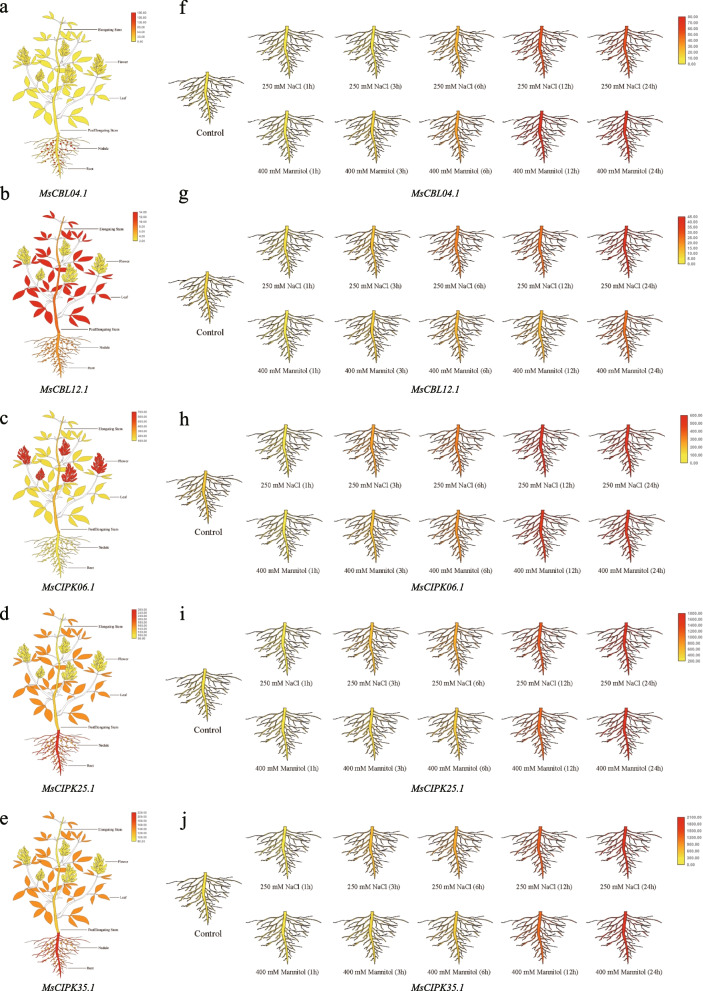
The expression profile of *CBL* and *CIPK* genes in *M. sativa* spp. *sativa*. **a-e** The expression of 2 *CBL*s and 3 *CIPK*s in different tissues. Six tissues are tested, including roots, mature leaves, developing flowers, elongating stem internodes, post-elongating stem internodes, and nitrogen fixing nodules. **f-j.** The expression of 2 *CBL*s and 3 *CIPK*s in roots under salt and drought stress. The roots were treated with by 250 mM NaCl or 400 mM mannitol

The *CBL*s and *CIPK*s are ubiquitously involved in developmental processes and responses to abiotic stress in plants [1, 54]. Our results show that *MsCBL*s and *MsCIPK*s are wildly expressed in various tissues from *M. sativa* spp. *sativa*, including roots, mature leaves, developing flowers, and nitrogen fixing nodules (Fig. 6), suggesting their critical roles in plant growth and development. Potassium is a fundamental macronutrient, which is involved in many physiological processes in plants [65, 66]. The Ca^2+^-mediated CBL-CIPK complex directly modulates activity of the plasma membrane-localized K^+^ channel to maintain K^+^ homeostasis. In *A. thaliana*, AtCBL4 and AtCIPK6 function in this process by regulating the activity of K^+^ channel, AKT2. Loss-of-function mutants exhibit reduced rosette size and delayed flowering [65, 66]. According to the phylogeny, *MsCBL04.1* and *MsCBL12.1*, duplicated by PWGD, are close to *AtCBL4*/*SOS3*, and *MsCIPK06.1* shares the highest similarity to *AtCIPK6*. Therefore, it can be assumed that *MsCBL04.1*, *MsCBL12.1* and *MsCIPK06.1* may play similar role in plant development by regulation of K^+^ balance. The different expression patterns of duplicate gene pair (*MsCBL04.1*/*MsCBL12.1*) indicate that a functional diversification may have occurred after PWGD ca. 55 million years ago [51].

*MsCIPK25.1* and *MsCIPK35.1* form a tandem duplicate gene pair (Additional file 7: Fig. S2) with the similar expression pattern. Their orthologous gene in *A. thaliana*, *AtCIPK25*, is highly expressed in roots (Fig. 6g, i), governing the root meristem size [67]. The close phylogenetic relationship of *MsCIPK25.1* and *MsCIPK35.1* indicates that this gene pair was generated by a recent tandem duplication, but hereditary changes have not been accumulated enough to drive the functional diversification of these two genes during the evolution of *M. sativa* spp. *sativa*.

Salt and drought are major environmental challengers that limit plant growth and development, and crop production. *CBL*s and *CIPK*s are recruited to cope with salt and drought stress in land plants [3, 68]. Generally, root is the crucial organ for plants to sense and respond to stress. In *A. thaliana,* AtCBL4/SOS3 activates AtCIPK24/SOS2 to launch the SOS pathway maintaining the ion homeostasis in roots under salt stress [45, 69]. This pathway has been widely reported to mediate the salt-stress response in rice, maize and sugarcane [19, 34, 46–48]. Moreover, CIPK6 and CIPK25 are key factor to confer the resistance to salt and drought stress in *A. thaliana* and *Gossypium hirsutum* [50, 70, 71]. Our results show that their orthologous genes in *M. sativa* spp. *sativa*, such as *MsCBL04.1*/*MsCBL12.1*, *MsCIPK25.1*/*MsCIPK35.1*, and *MsCIPK06.1*, also are highly induced in roots under salt and drought stress conditions, suggesting that these genes act as positive regulators in response to salt and drought stress, probably via modulating the ion homeostasis. Taken together, 2 *CBL* genes and 3 *CIPK* genes may play key roles in response to salt and drought stress in *M. sativa* spp. *sativa*, and further studies should be performed for in-depth understanding of their functions in stress tolerance.

## Conclusions

In summary, we identify 68 *CBL* and 135 *CIPK* genes from 5 *Medicago* species/subspecies at the haploid genome level. PWGD, Msa-WGD, and small-scale tandem duplication have contributed to the expansion of both gene families in *Medicago*. The gene structure, protein motif, syntenic relationship and phylogenomic relationship of both *CBL* and *CIPK* gene families are relatively conserved, shedding new lights on the evolution of *CBL* and *CIPK* gene families in *Medicago*. Furthermore, five genes, including 2 *MsCBL*s and 3 *MsCIPK*s, may play critical roles in response to salt and drought stress as well as in various developmental processes. Thus, our study provides candidates for improvement of cultivated *M. sativa* with enhanced tolerance to salt and drought stress by molecular breeding approaches.

## Methods

### Identification of *CBL* and *CIPK* genes in *Medicago*

The *M. truncatula* and *C. arietinum* genomes were downloaded from Phytozome V13 (https://phytozome-next.jgi.doe.gov/), and the genomes for *M. sativa* ssp. *sativa, M. sativa* ssp. *caerulea*, *M. polymorpha*, and *M. ruthenica* were obtained by following the previous studies [22, 23, 25, 27]. Ten CBL and 26 CIPK protein sequences from *A. thaliana* were queried against the protein annotation databases of selected six genomes via local BLASTp with e-value < 1E-5 and identity > 90% [10]. The candidate sequences were first evaluated by NCBI conserved domain search tools (https://www.ncbi.nlm.nih.gov/Structure/cdd/wrpsb.cgi), and then manually verified by conserved motifs. All CBL proteins were identified by the four EF-hand motifs. Similarly, a conserved NAF/FISL motif was also characterized for all CIPK proteins. The identified protein sequences were used to compute protein MW and pI via Expasy tools with default set (https://web.expasy.org/protparam/).

### Phylogeny and classification of *CBL* and *CIPK* gene families

The multiple protein sequences of identified CBL and CIPK families were aligned using MUSCLE v.3.8.31 software with default parameters [72]. The resulted alignments of CBL and CIPK sequences were trimmed via trimAL1.4 with the automated1 parameter respectively [73]. The trimmed alignments were used to construct the maximum likelihood (ML) phylogenetic trees using RAxML v.8.2.9 with the PROTGAMMALGX model for 1000 bootstrap replicates [74]. The ML trees were visualized using Interactive Tree of Life (iTOL) (https://itol.embl.de/). *C. arietinum* and *G. max* were selected as outgroup species, which share the PWGD event with *Medicago* species. The *CBL*s and *CIPK*s from *C. arietinum* and *G. max* were chosen for identification of duplicate genes generated by the PWGD. Furthermore, *G. max* is a tetraploid species, while *C. arietinum* is a diploid species. *Medicago* contains both tetraploid and diploid plants. Therefore, *G. max* and *C. arietinum* are essential to investigate the preference of duplication and preservation in diploids, allotetraploids, and autotetraploids from different lineages of Papilionoideae.

### Chromosomal location, gene structure, and conserved motif pattern investigation

From the downloaded general feature files of six genomes, we obtained the chromosomal location and gene structure information of *CBL* and *CIPK* genes, which were further illustrated using TBtools [75]. The conserved motifs of each CBL and CIPK protein were further confirmed using MEME program (https://meme-suite.org/meme/tools/meme). The conserved motif sequences were demonstrated as Seq Logos via TBtools [75], and the iTOL was used to display the MEME discoveries and gene structures in the circular mode.

### Synteny analysis and *Ks* calculation

The synteny analysis was performed by intra/interspercies compairisons using MCScanX [76]. The duplicate gene pairs generated by tandem duplication and WGD were retieved based on the synteny analysis. The WGD-derived duplicate gene pairs were subject to PAML for calculation of *Ks* values [77]. *Ks* values were used to set apart the duplicate gene pairs produced by the autotetraploid WGD event in *M. sativa* ssp. *sativa* from those generated by the common WGD event shared by all Papilionoideae species. The *Medicago* species and *C. arietinum* shared the *Ks* value around 0.62 [26], which indicates the occurrence of WGD event in the common ancestors of Papilionoideae. *C. arietinum* was selected as out group to detect the duplicate gene pairs from the PWGD or Msa-WGD by *Ks* values.

### Expression profiling of *CBL* and *CIPK* genes in *M. sativa* spp. *sativa*

The RNA-seq raw data of *M. sativa* spp. *sativa* was obtained from previous studies [28, 29], which contains two parts: part I is associated with six different tissues, including roots, mature leaves, developing flowers, elongating stem internodes, post-elongating stem internodes, and nitrogen fixing nodules; part II is collected from roots treated by NaCl (salt stress) or mannitol (drought stress) after 1, 3, 6, 12 and 24 h respectively. After strict quality evaluation, clean reads were re-analyzed with the reference genome of *M. sativa* spp. *sativa* [23] via Bowtie2 [78]. Gene expression was quantified by fragments per kilobase per million (FPKM) using RSEM package [78]. The expression patterns were visualized via TBtools [75].

### Plant growth conditions and stress treatment

The *M. sativa* spp. *sativa* accession ‘Zhongmu No. 1 was grown at 22 ℃ under long-day conditions (16 h light: 8 h dark). The seeds were sterilized in 3% H_2_O_2_ for 10 min, then washed twice with sterile water. The seeds were placed on moist filter paper in Petri dishes for 5 days. The seedlings exhibiting similar growth were selected and cultured in half-strength Murashige and Skoog (1/2 MS) liquid media (PH 5.8). Seven days post transplantation, the seedlings were exposed to 1/2 MS liquid media supplemented with 250 mM NaCl or 400 mM mannitol. Root samples were carefully harvested 1 h, 3 h, 6 h, and 12 h after treatment respectively.

### RNA extraction and quantitative RT-PCR analysis

Around 100 mg of fresh root sample was ground frozen via a Tissue Lyer II homogenizer (Qiagen). Total RNA was extracted using PrimeSciptTM reagent Kit (Takara, Japan) following the manufacture’s instruction. DNA contamination was removed by RQ1 RNase-Free DNase (Promega). cDNA synthesis was performed using SuperScriptII reverse transcriptase (Thermo Fisher Scientific) with oligo (dT) primers. qRT-PCR was performed with SYBR Green Supermix (Takara, Japan) in a CFX96 Real Time System (Bio-Rad). The PCR reaction was carried out as follows: 95 ℃ denaturation for 30 s, followed by 40 cycles of 94 ℃ for 10 s, 60 ℃ for 10 s, and 72 ℃ for 10 s. Primes used in this study were listed in Additional file 14 and Table S6.

## Supplementary Information

**Additional file 1: Table S1.** Summary of *CBL* gene characteristics in *Medicago* and *C.* *arietinum*.

**Additional file 2: Table S2.** Summary of *CIPK* gene characteristics in *Medicago* and *C.* *arietinum*.

**Additional file 3: Figure S1. **Chromosomal localization of the *CBL* and *CIPK* genes in *M. sativa* spp. *sativa*.

**Additional file 4: Table S3. **CBL protein sequences used to build phylogenetic tree.

**Additional file 5: Figure S2. **Subcellular localization motifs of CBL proteins at the N-terminus.

**Additional file 6: Table S4. **CIPK protein sequences used to build phylogenetic tree.

**Additional file 7: Table S5. Ks** value of paralog pairs of *CBL *and *CIPK* genes in *M. sativa* spp. *sativa*.

**Additional file 8: Figure S3.** Synteny analysis of *CBL* and *CIPK* genes among four allelic chromosomes in *M. sativa* spp. *sativa*. *CBL* genes are in red, and *CIPK* genes are in gray. Red lines represent the collinearity of duplicated *CBL* genes, and gray lines indicate the collinearity of duplicated *CIPK* genes. Four sets of chromosomes in chromosome circles are represented by four different colors, light green for the first set of chromosomes (MsaX.1), light blue for the second set of chromosomes (MsaX.2), pick for the third set of chromosomes (MsaX.3), and orange for the fourth set of chromosomes (MsaX.4). MsaX indicates chromosome 1 to 8.

**Additional file 9: Figure S4. **Expression of *CBL* genes in different tissues from *M. sativa* spp. *sativa*.

**Additional file 10: Figure S5.** Expression of *CIPK *genes in different tissues from *M. sativa* spp. *sativa*.

**Additional file 11: Figure S6.** Expression of *CBL* genes in roots of *M. sativa* spp. *sativa* when exposed to salt and drought stress.

**Additional file 12: Figure S7.** Expression of *CIPK* genes in roots of *M. sativa* spp. *sativa* when exposed to salt and drought stress.

**Additional file 13: Figure S8.** Expression analysis of selected genes by qRT-PCR in roots from* M. sativa* spp. *sativa* exposed to salt and drought stress. Plants were treated with 250 mM NaCl or 400 mM mannitol, and roots were carefully harvested 1h, 3h, 6h, and 12h after treatments respectively. *MsUBQ* gene was used as internal control. The experiments were performed in triplicates with a representative result displayed, and values are the mean ± SE. The lowercase letters indicate significant difference (Tukey’s multiple comparison test, *p* < 0.05).

**Additional file 14: Figure S9. **The protein interaction network between CBLs and CIPKs in* A. thaliana* and *M. sativa* spp.* sativa*. The interaction network in *M. sativa* spp.* sativa *was predicted based on gene co-expression.

**Additional file 15: Table S6. **The primers used in this study.

## Data Availability

All data analyzed in this study are included within the article and attached to the Additional files.

## Abbreviations

CBL: Calcineurin B-like protein
CIPK: CBL-interacting protein kinase
TTS: Tonoplast targeting sequence
TM helix: Transmembrane helical region
ABI: Abscisic acid insensitive
PP2C: Protein phosphatase 2C
SOS: Salt overly sensitive
ML: Maximum-likelihood
WGD: Whole genome duplication
PWGD: The ancestral Papilionoideae whole-genome duplication event
Msa-WGD: The lineage-specific autotetraploidy independent of WGD event in *M. sativa* spp. *sativa*
GBH: Gene balance hypothesis
