# Supplementary figures and images for: Evolution of the CBL and CIPK gene families in Medicago: genome-wide characterization, pervasive duplication, and expression pattern under salt and drought stress
Source: BMC Plant Biol. 2022 Nov 3;22:512. doi: 10.1186/s12870-022-03884-3 (PMC9632064; doi:10.1186/s12870-022-03884-3)

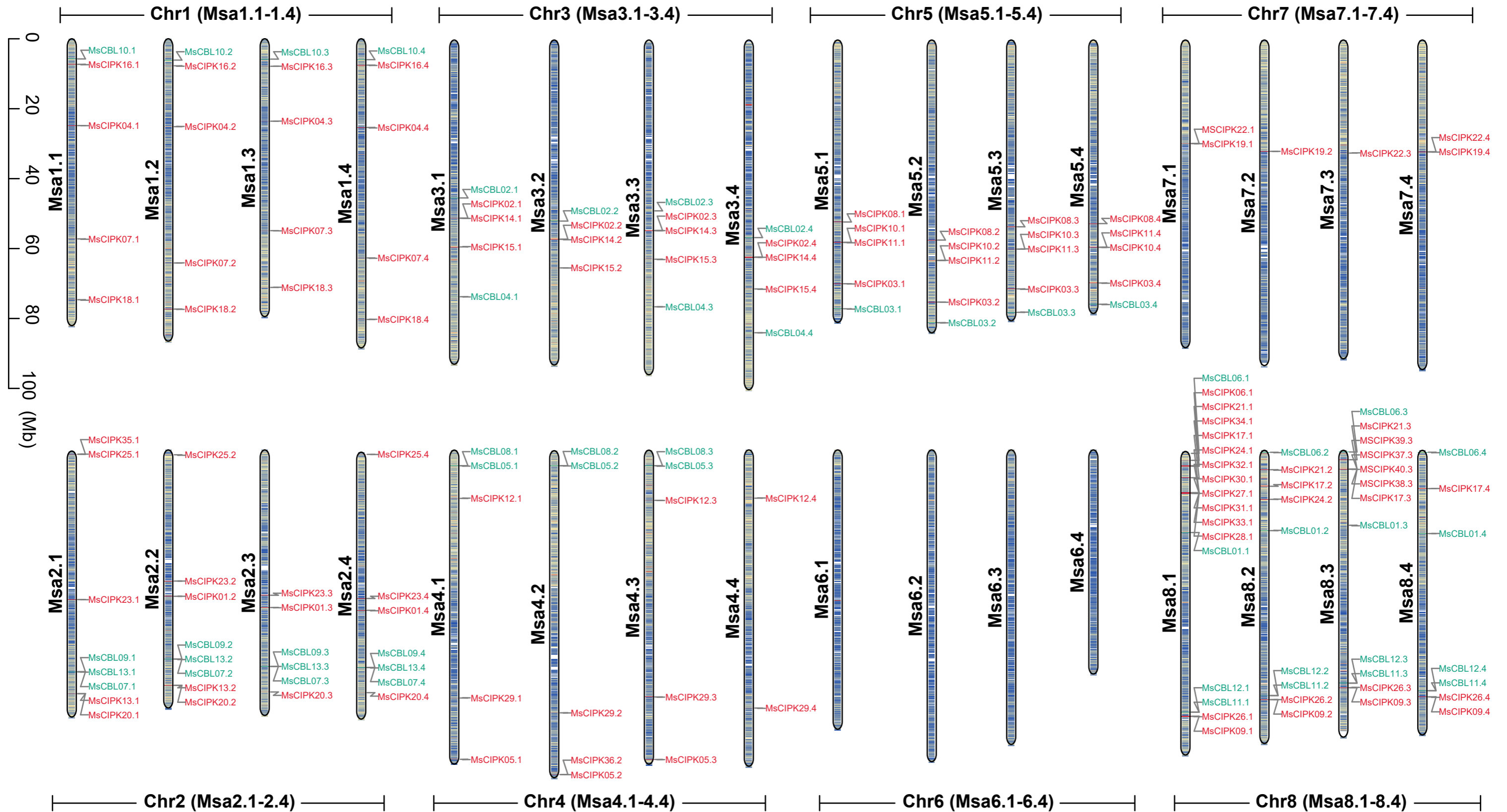

Supplement: Supplementary file 3 — Additional file 3: Figure S1. Chromosomal localization of the CBL and CIPK genes in M. sativa spp. sativa. [file 12870_2022_3884_MOESM3_ESM.pdf]

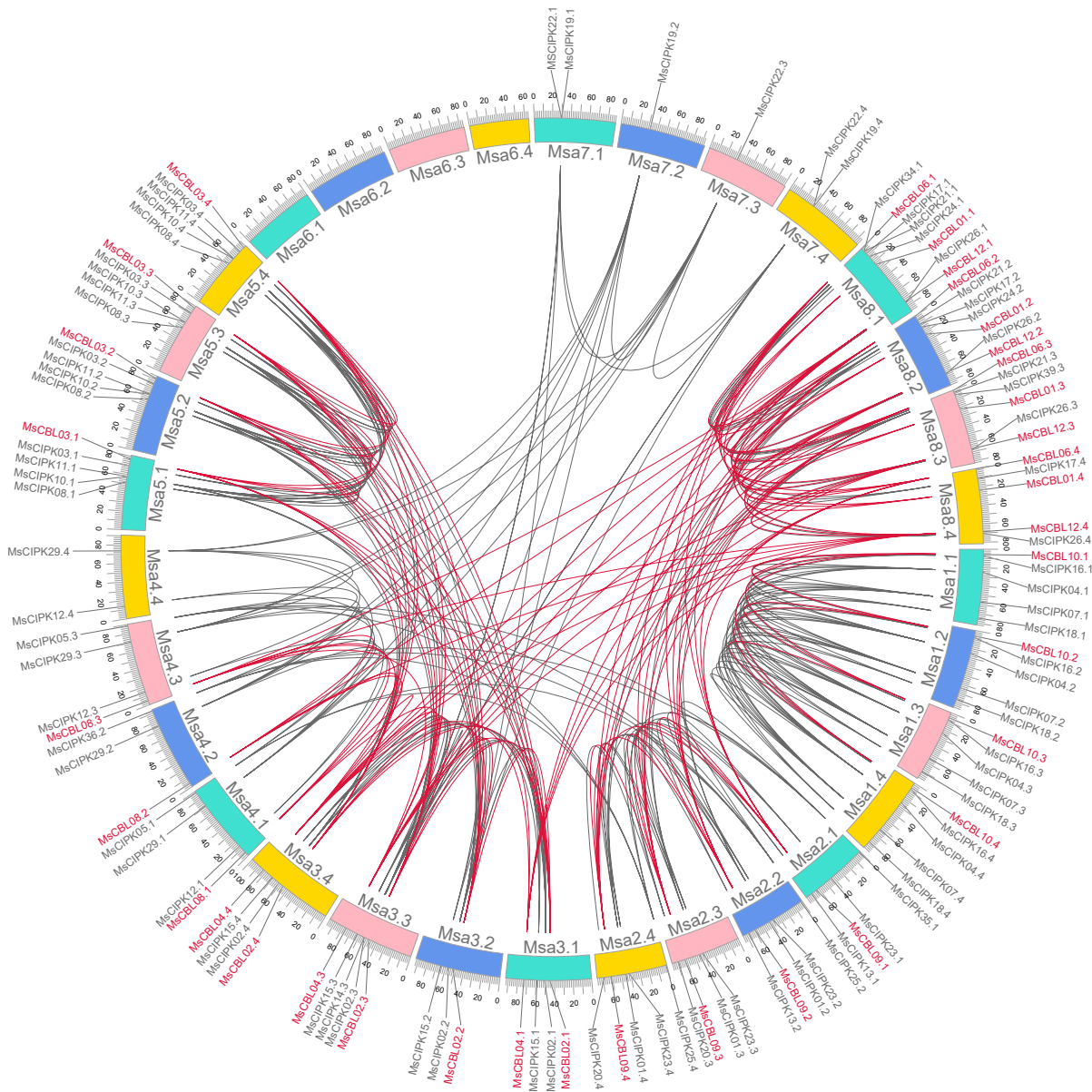

Supplement: Supplementary file 8 — Additional file 8: Figure S3. Synteny analysis of CBL and CIPK genes among four allelic chromosomes in M. sativa spp. sativa. CBL genes are in red, and CIPK genes are in gray. Red lines represent the collinearity of duplicated CBL genes, and gray lines indicate the collinearity of duplicated CIPK genes. Four sets of chromosomes in chromosome circles are represented by four different colors, light green for the first set of chromosomes (MsaX.1), light blue for the second set of chromosomes (MsaX.2), pick for the third set of chromosomes (MsaX.3), and orange for the fourth set of chromosomes (MsaX.4). MsaX indicates chromosome 1 to 8. [file 12870_2022_3884_MOESM8_ESM.pdf]

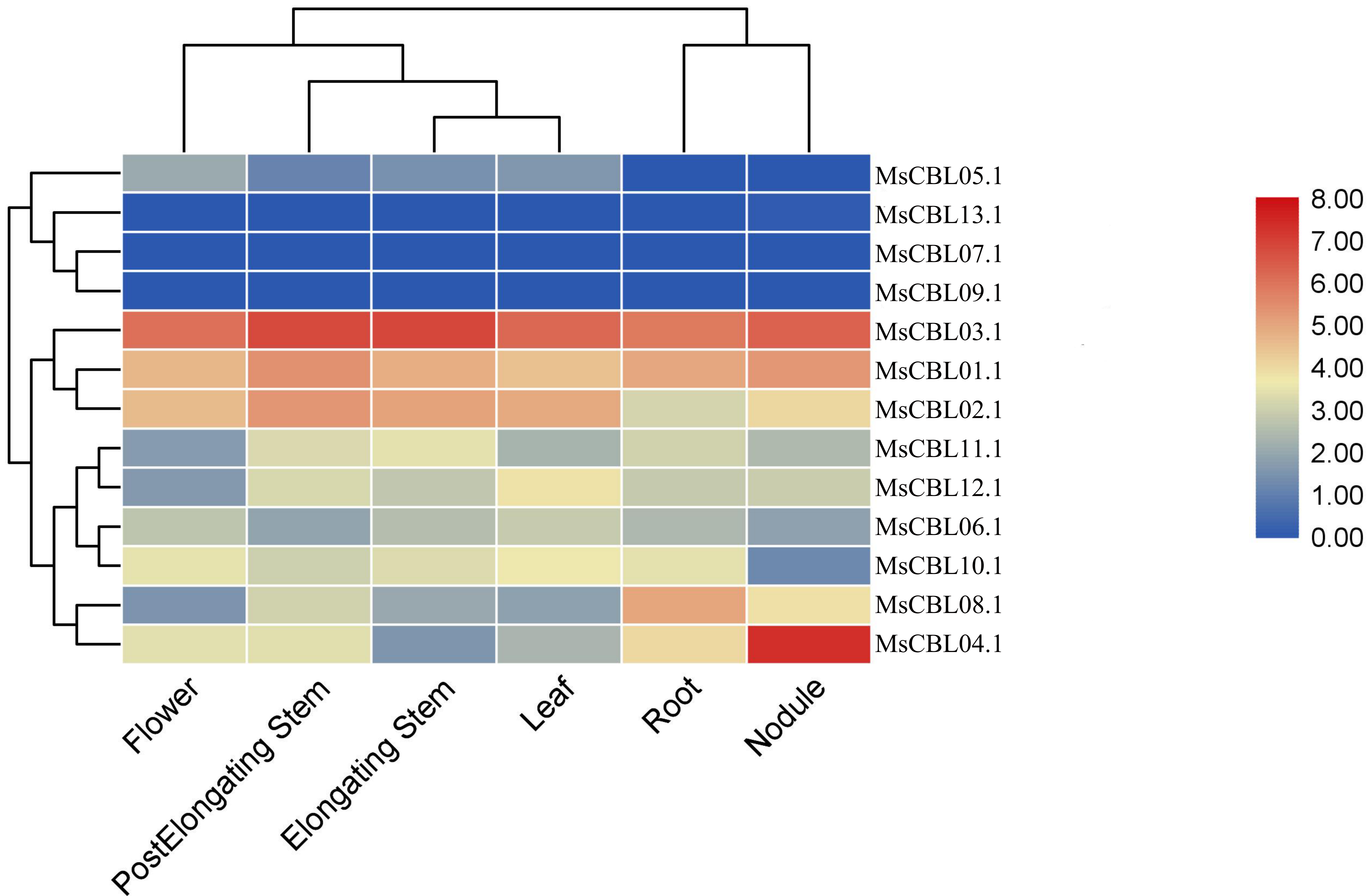

Supplement: Supplementary file 9 — Additional file 9: Figure S4. Expression of CBL genes in different tissues from M. sativa spp. sativa. [file 12870_2022_3884_MOESM9_ESM.pdf]

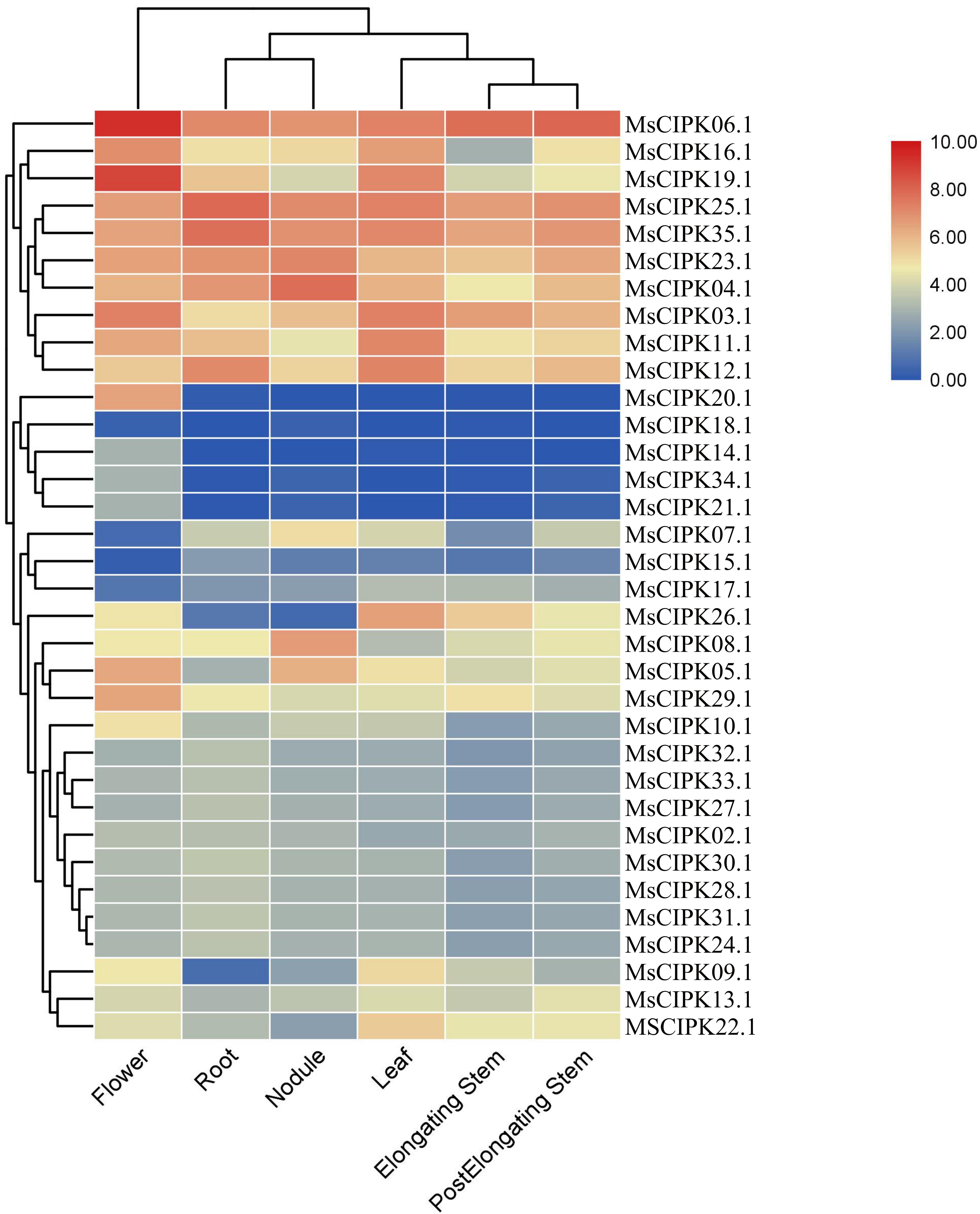

Supplement: Supplementary file 10 — Additional file 10: Figure S5. Expression of CIPK genes in different tissues from M. sativa spp. sativa. [file 12870_2022_3884_MOESM10_ESM.pdf]

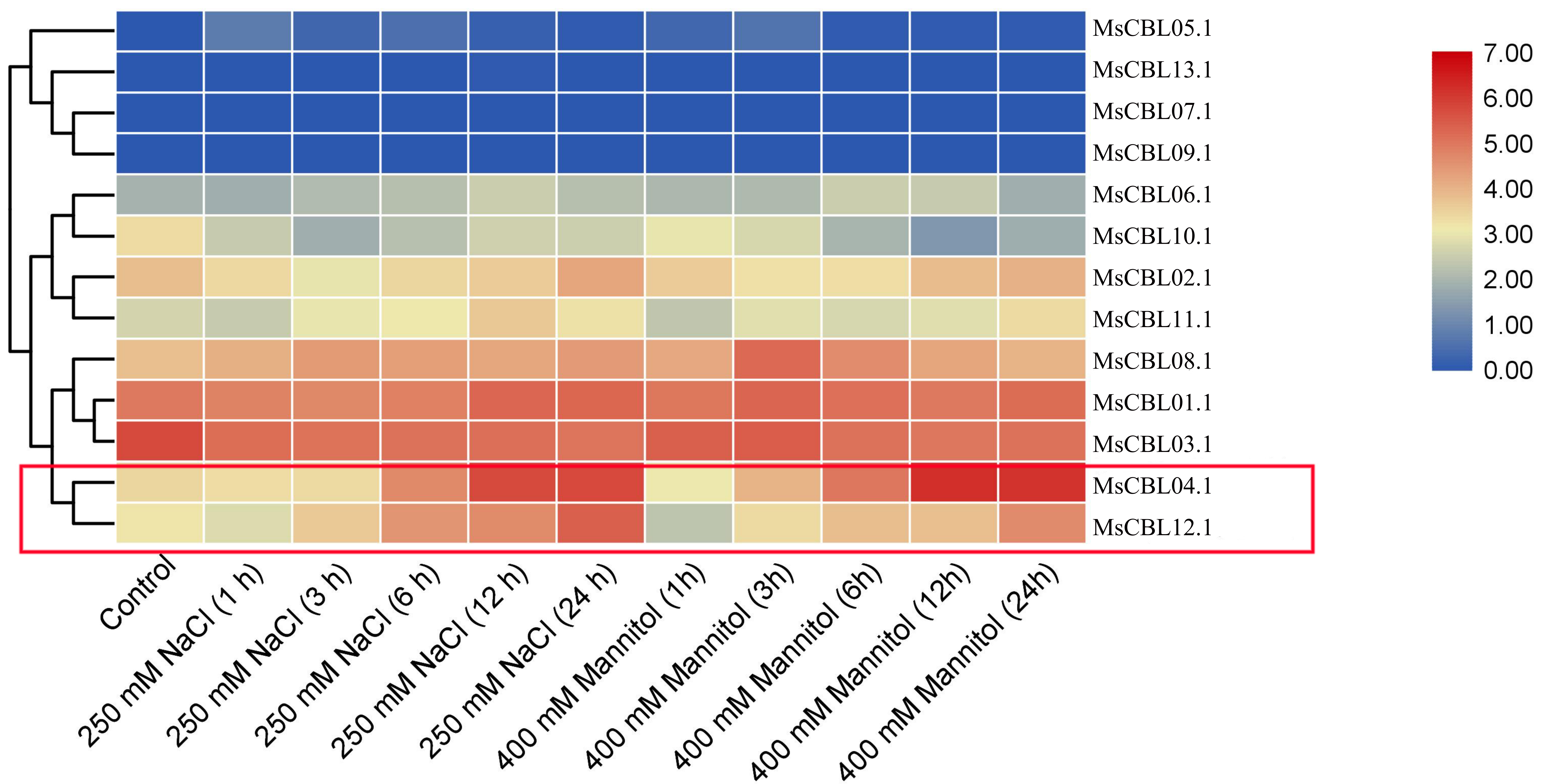

Supplement: Supplementary file 11 — Additional file 11: Figure S6. Expression of CBL genes in roots of M. sativa spp. sativa when exposed to salt and drought stress. [file 12870_2022_3884_MOESM11_ESM.pdf]

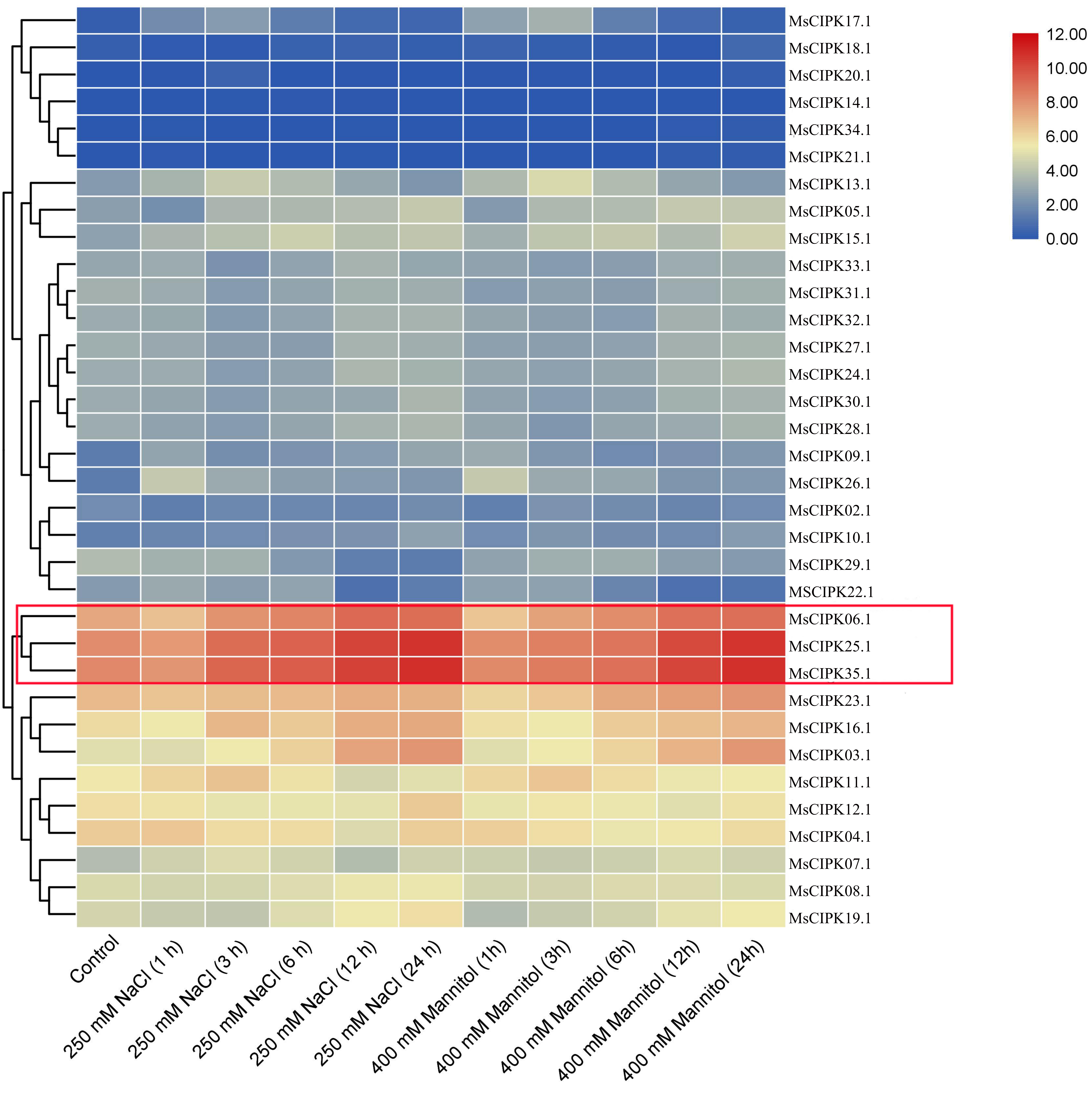

Supplement: Supplementary file 12 — Additional file 12: Figure S7. Expression of CIPK genes in roots of M. sativa spp. sativa when exposed to salt and drought stress. [file 12870_2022_3884_MOESM12_ESM.pdf]

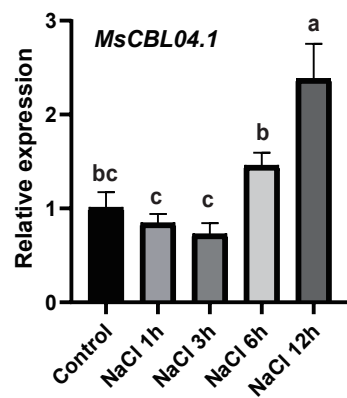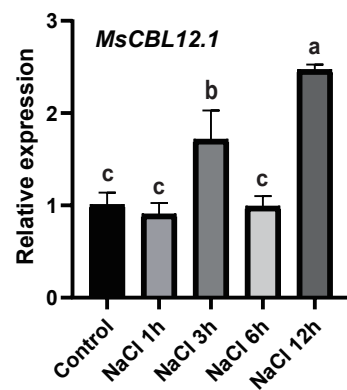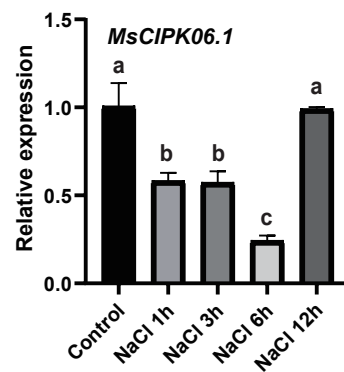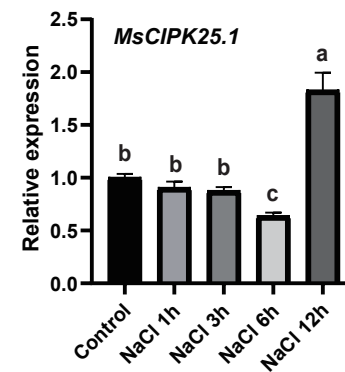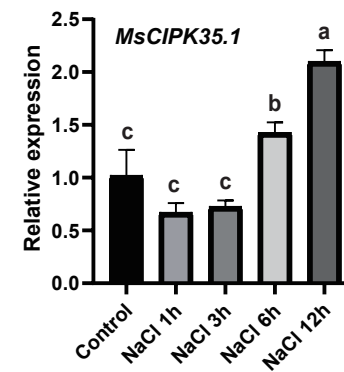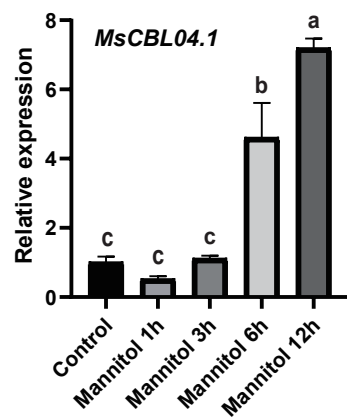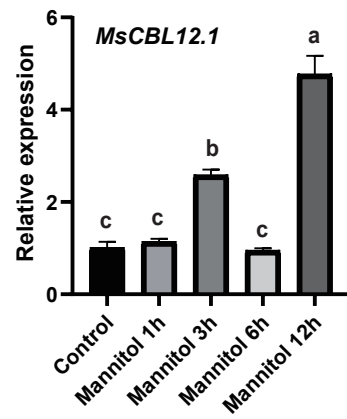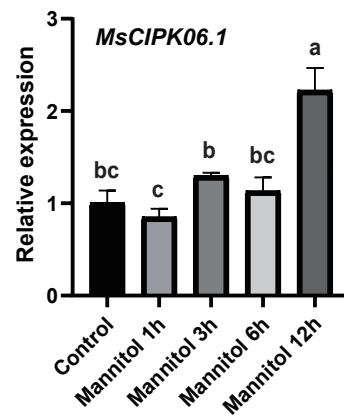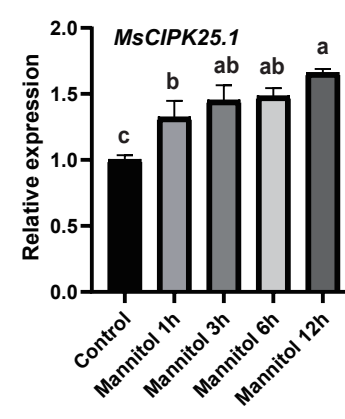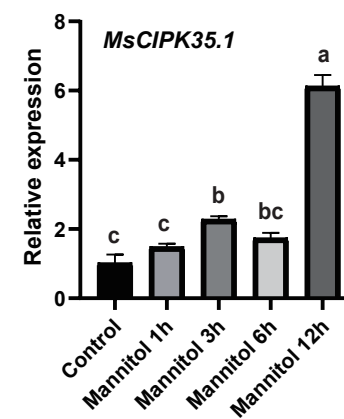

Supplement: Supplementary file 13 — Additional file 13: Figure S8. Expression analysis of selected genes by qRT-PCR in roots from M. sativa spp. sativa exposed to salt and drought stress. Plants were treated with 250 mM NaCl or 400 mM mannitol, and roots were carefully harvested 1h, 3h, 6h, and 12h after treatments respectively. MsUBQ gene was used as internal control. The experiments were performed in triplicates with a representative result displayed, and values are the mean ± SE. The lowercase letters indicate significant difference (Tukey’s multiple comparison test, p < 0.05). [file 12870_2022_3884_MOESM13_ESM.pdf]

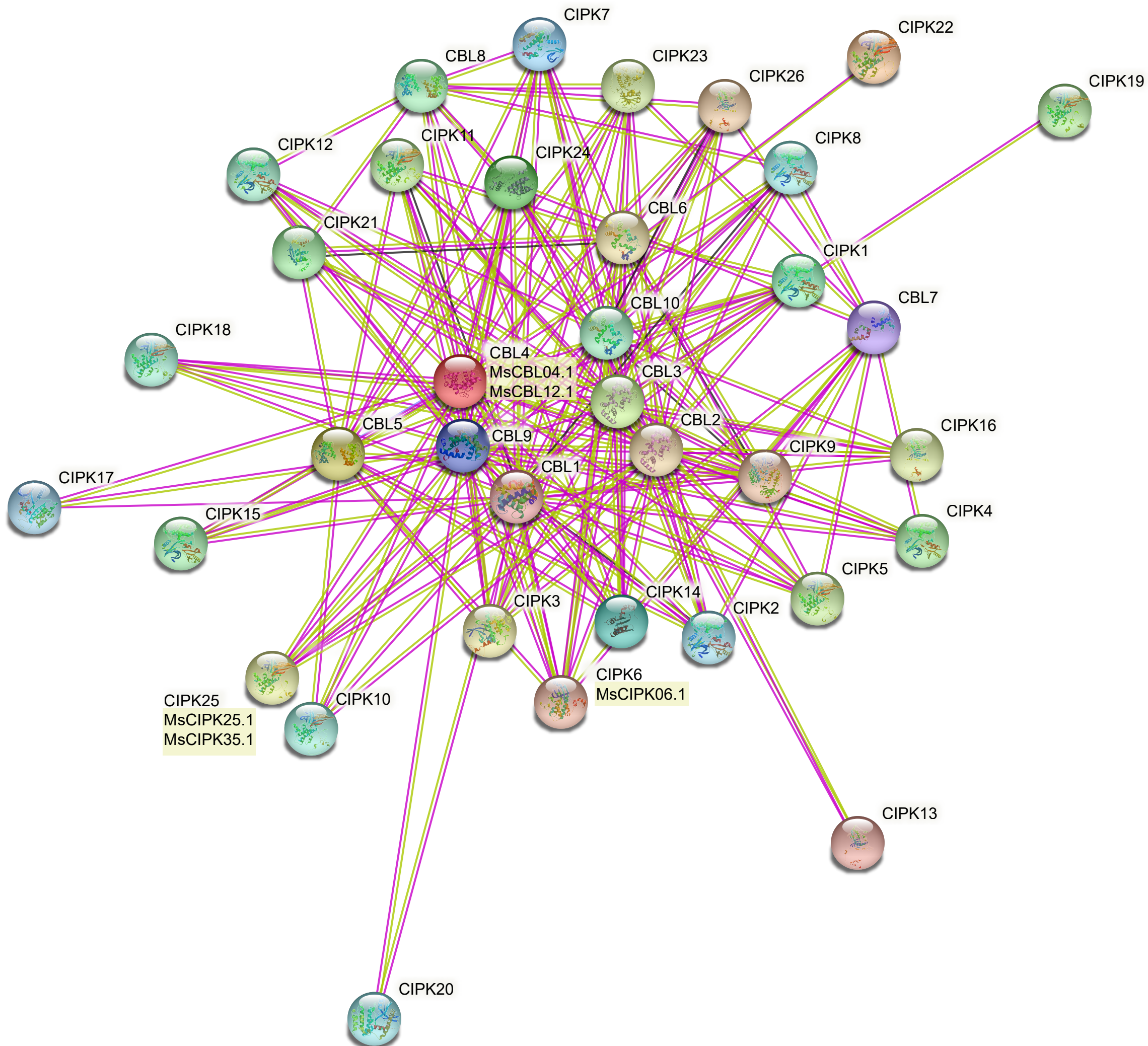

Supplement: Supplementary file 14 — Additional file 14: Figure S9. The protein interaction network between CBLs and CIPKs in A. thaliana and M. sativa spp. sativa. The interaction network in M. sativa spp. sativa was predicted based on gene co-expression. [file 12870_2022_3884_MOESM14_ESM.pdf]
