# Supplementary material for: Evolution of the CBL and CIPK gene families in Medicago: genome-wide characterization, pervasive duplication, and expression pattern under salt and drought stress
Source: BMC Plant Biol. 2022 Nov 3;22:512. doi: 10.1186/s12870-022-03884-3 (PMC9632064; doi:10.1186/s12870-022-03884-3)

Type I CBL proteins: Dual lipid modification motif

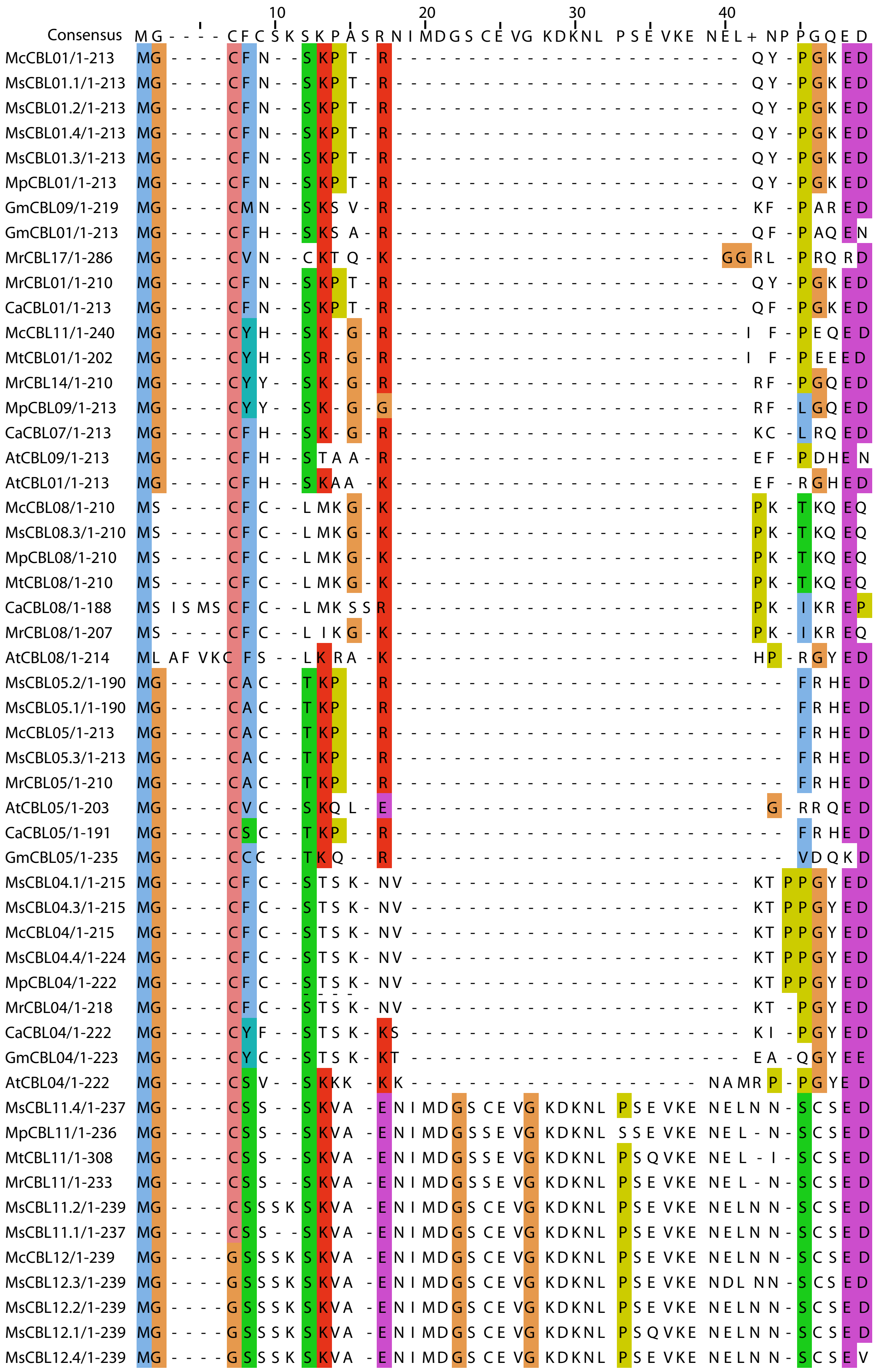

Type II CBL proteins: Tonoplat targeting sequence (TTS)

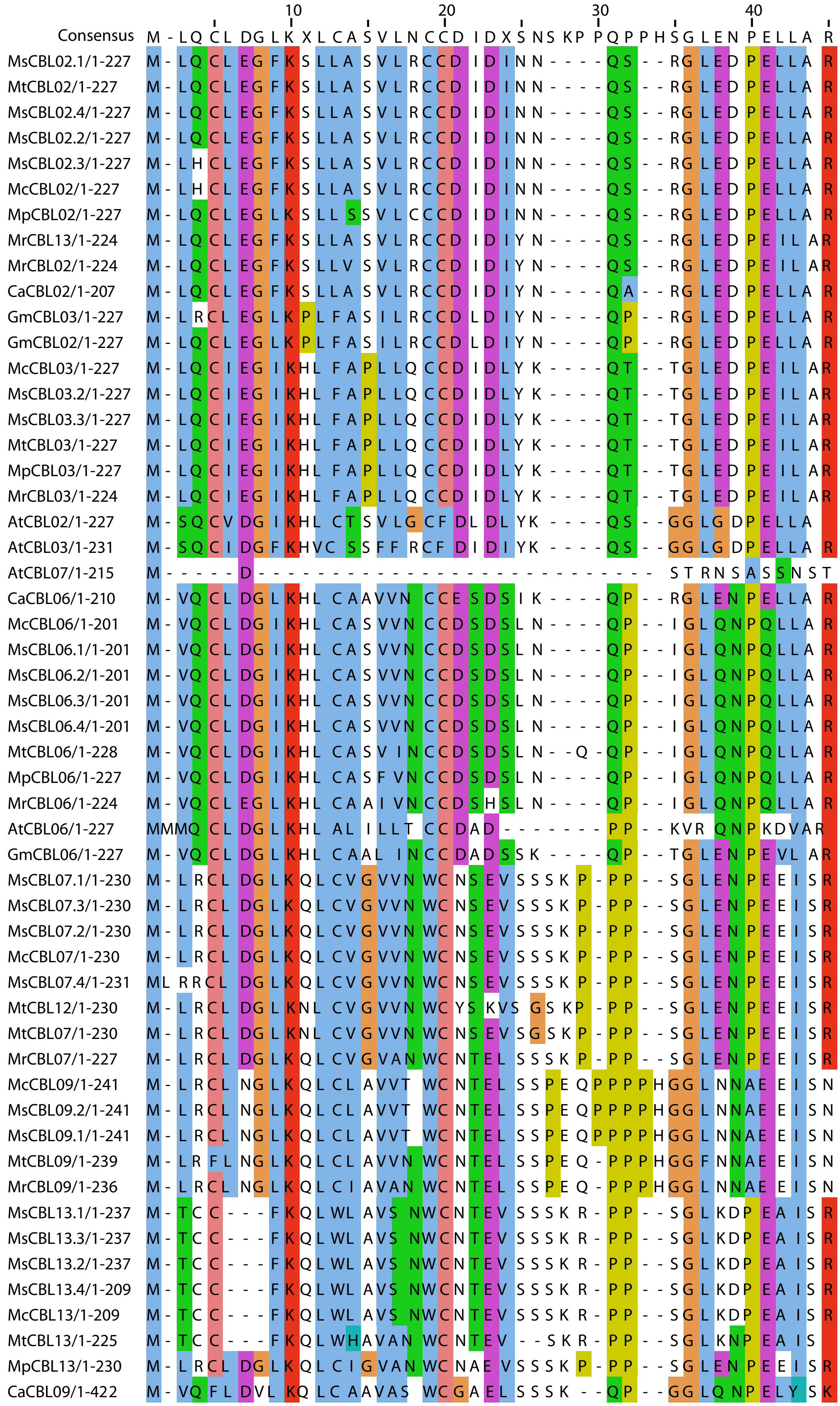

Type III CBL proteins: transmembrane helical region (TM helix)

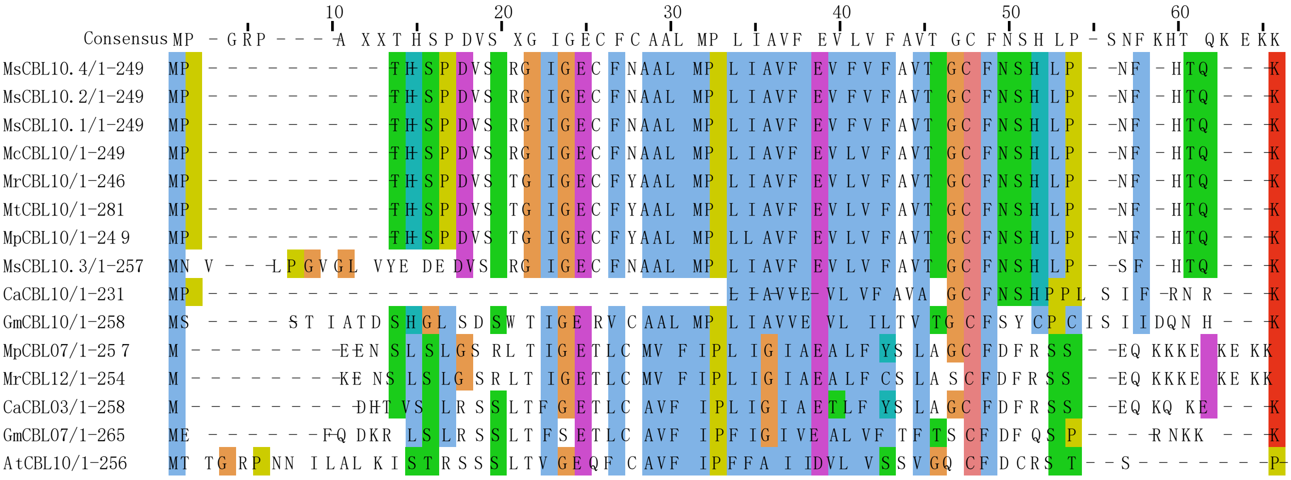

Supplement: Supplementary file 5 — Additional file 5: Figure S2. Subcellular localization motifs of CBL proteins at the N-terminus. [file 12870_2022_3884_MOESM5_ESM.pdf]
